# Survival outcomes in prostate cancer patients treated at an Indian tertiary care centre

**DOI:** 10.1002/bco2.70155

**Published:** 2026-02-05

**Authors:** Aswathy P, Sivaranjini Kannusamy, Amey Oak, Gagan Prakash, Amit Joshi, Vedang Murthy, Santosh Menon, Sandhya Cheulkar, Monika Lokhande, Ganesh Balasubramaniam, Rajesh Dikshit, Pankaj Chaturvedi, Sudeep Gupta

**Affiliations:** ^1^ Homi Bhabha National Institute Mumbai India; ^2^ Advanced Centre for Treatment, Research and Education in Cancer (ACTREC) Mumbai India; ^3^ Centre for Cancer Epidemiology Mumbai India; ^4^ Tata Memorial Centre Mumbai India; ^5^ Division of Cancer Care, Hospital Cancer Registries & Survival Studies Centre for Cancer Epidemiology Mumbai India; ^6^ Department of Surgical Oncology Mumbai India; ^7^ Department of Medical Oncology Mumbai India; ^8^ Department of Radiation Oncology Mumbai India; ^9^ Department of Pathology Mumbai India

**Keywords:** Gleason grade, prostate cancer, PSA level, survival

## Abstract

**Background:**

Prostate cancer is an emerging public health concern in India, with rising incidence and varying survival outcomes. This study aimed to evaluate 5‐year overall survival and identify prognostic factors among prostate cancer patients treated at Tata Memorial Hospital (TMH), Mumbai.

**Methods:**

This retrospective study included all patients newly diagnosed with prostate cancer between January and December 2017 who received cancer‐directed treatment at TMH. Patients were followed through 2022. Clinico‐epidemiological variables including age, PSA levels, Gleason grade, clinical extent (EAU risk group classification), intent, completion status and treatment modality were analysed. Kaplan–Meier survival curves and Cox proportional hazards models were used to assess survival outcomes.

**Results:**

A total of 421 patients were included, with a mean (SD) age of 66 ± (8.39) years and a median (IQR) PSA of 45.9 (17–154) ng/ml. All patients were symptomatic at presentation, predominantly with urinary complaints (90.5%), followed by bone pain (3.6%) or both (5.9%). At diagnosis, 15.2% had localized disease, 25.8% had locally advanced disease, and 58.4% had metastatic cancer. The overall 5‐year survival rate was 61%. Prognostic factors significantly associated with survival included age, PSA, Gleason grade and disease extent. Patients with PSA > 1000 ng/ml had the poorest prognosis (33% 5‐year survival). Survival varied by age group, declining from 66% in those aged 66–75 years to 45% in patients >75 years, although this trend was not statistically significant in adjusted analysis (*p* = 0.46). Disease extent demonstrated a strong survival gradient: 89% in localized disease, 79% in locally advanced and 41% in metastatic cancer, with metastatic disease showing a significantly increased adjusted mortality risk (HR: 4.59; *p* 0.004). Curative treatment intent was associated with markedly better outcomes, with a 5‐year survival of 81% compared to 40% among those receiving palliative care. Similarly, treatment adherence had a substantial impact on prognosis, with patients completing therapy achieving a 65% 5‐year survival rate, in contrast to only 12% among those with incomplete treatment (adjusted HR: 3.62; *p* < 0.001). Treatment modality also influenced survival: patients treated with ADT alone had the lowest 5‐year survival (35%) and a significantly higher mortality risk (adjusted HR:1.65; *p* 0.01), whereas outcomes were more favourable with radical prostatectomy with adjuvant therapy (75%; adjusted HR: 1.21 *p* 0.80).

**Conclusion:**

Survival in prostate cancer is strongly influenced by clinical extent at diagnosis, PSA, Gleason grade and treatment. Improving early detection, expanding multimodal treatment strategies and ensuring treatment completion are critical to enhancing outcomes in India.

## 1 INTRODUCTION

Prostate cancer is one of the most prevalent malignancies affecting men, particularly in the elderly population.^1^ In 2022, the global landscape revealed alarming statistics: 1466 750 new diagnoses and 396 987 deaths, making it the second most diagnosed cancer and the fifth leading cause of cancer‐related mortality among males.^2^ In South‐Central Asia, India alone accounts for 60% of the region's prostate cancer cases, with an age‐standardized incidence rate of 5.6 per 100 000 individuals and a mortality rate of 2.7 per 100 000. In India, prostate cancer incidence has shown a consistent upward trend over the past three decades.^2^ Data from six Population‐Based Cancer Registries (PBCRs) between 1982 and 2016 indicate annual average percentage changes (AAPC) ranging from 1.5% in Mumbai to 4.7% in Chennai.^3^ This rising incidence is attributed to increasing life expectancy, urbanization, lifestyle changes, improved health literacy and greater access to diagnostic facilities, including prostate‐specific antigen (PSA) testing.^4^

The diagnostic profile in India is strikingly different from that in Western countries. In the United States and Europe, widespread PSA screening has led to higher incidence and detection of disease at earlier, often asymptomatic stages.^5^, ^6^ In India, however, there is no national screening programme and population‐wide PSA testing due to concerns about overdiagnosis.^7^, ^8^ Consequently, most prostate cancers in India are detected symptomatically, often when men present with lower urinary tract complaints or bone pain, prompting PSA testing rather than being identified through proactive screening of asymptomatic individuals.^9^ An estimated 80%–85% of Indian patients present at stage III or IV, compared to ~15% in the United States.^6^ These late‐stage presentations contrast sharply with Western cohorts, where PSA‐driven surveillance has shifted diagnosis to organ‐confined disease.^10^

Treatment practices closely follow diagnostic patterns. In Europe and North America, radical prostatectomy and radiotherapy constitute the standard of care for localized prostate cancer. In India, however, androgen deprivation therapy (ADT) remains the predominant treatment, largely due to late‐stage presentation, while curative modalities are underutilized.^11^, ^12^, ^13^ This treatment gap translates into markedly poorer survival outcomes. A hospital‐based study from Tata Memorial Hospital (TMH) reported a 5‐year overall survival of 64%, whereas a population‐based study from the Sangrur and Mansa registries documented only 30.3%.^14^, ^15^ In contrast, SEER data from the United States show a 5‐year relative survival of 97.1%, with near‐universal survival among patients with localized disease.^16^

This study, carried out at TMH in Mumbai, a leading centre for cancer treatment and research in India focused on estimating the 5‐year overall survival rate for prostate cancer patients. The study also investigated how various clinico‐epidemiological factors impact overall survival. Utilizing data collected from 2017 and tracking patients through 2022, the research seeks to identify key prognostic factors that can inform public health strategies, enhance treatment protocols and provide crucial insights for patient counselling and education.

## 2 METHODOLOGY

The current study involved a retrospective analysis of hospital records from the TMH Cancer Registry. It included all patients newly diagnosed with primary prostate cancer between 1 January and 31 December 2017, who had received any form of cancer‐directed treatment at TMH. Data were collected using patient files, the hospital's electronic medical record (EMR) system and the Hospital‐Based Cancer Registry (HBCR) internal software. The study gathered demographic information such as age, education, occupation, income, region and clinical data, including primary histology classified by International Classification of Diseases for Oncology‐3 (ICD‐O‐3)^17^ coding, Prostate Specific Antigen levels and Gleason scores. The clinical extent of the disease was categorized using the standard European Association of Urology (EAU) classification^18^ (Table 1).

**TABLE 1:** Definition of clinical extent of the disease (European Association of Urology).

Definition of clinical extent of the disease (European Association of Urology)^18^			
Low risk	Intermediate risk	High risk	
PSA < 10 ng/ml	PSA 10–20 ng/ml	PSA > 20 ng/ml	Any PSA
And GS < 7	Or GS 7	Or GS > 7	Any GS
(ISUP grade 1)	(ISUP grade 2/3)	(IUSP grade 4/5)	(any IUSP grade)
and cT1‐2a	Or cT2b	cT2c	cT3–4 or CN+
Localized			Locally advanced

Additionally, information regarding treatment completion, intention of treatment and treatment modality was collected. For each patient, the start date was defined as the date of diagnosis, and the follow‐up period extended until 31 December 2022, or the date of death or loss to follow‐up, whichever occurred first. Follow‐up information was gathered from the EMR and through phone interviews conducted by the HBCR staff as part of their service. The study received ethical approval, with Institutional Review Board number 901028.

Data analysis was performed using STATA software version 15.0 (STATA‐CORPLLC, College Station, TX, USA).^19^ Continuous variables were presented as mean (SD) or median (IQR) depending on their distribution, while categorical variables were summarized as proportions. Overall survival was calculated using the Kaplan–Meier method, and the log‐rank test was employed to assess survival differences across various factors. The Cox proportional hazard model was applied to evaluate the simultaneous impact of multiple factors on survival. A *p*‐value of less than 0.05 was considered statistically significant. Data visualization was done using R software version 4.5.0.

## 3 RESULTS

**FIGURE 1:**
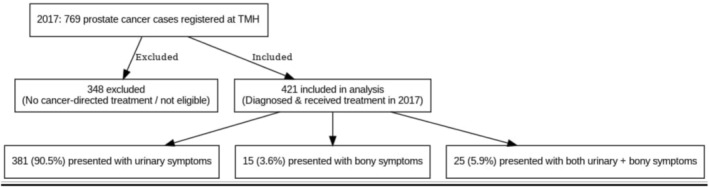
Distribution of symptoms at presentation of study participants (*n* = 421).

In 2017, a total of 769 prostate cancer cases were registered at TMH, of which 421 (54.7%) patients received cancer‐directed treatment and were included in this study, while the remainder were excluded due to not initiating treatment. The majority of patients presented with symptoms: 381 (90.5%) reported urinary complaints, 15 (3.6%) had bony symptoms, and 25 (5.9%) presented with both. As TMH is a tertiary cancer centre, all patients presenting are symptomatic and already have PSA values from outside centres at the time of referral. There were no asymptomatic individuals in our cohort. The flow of cases and their clinical presentation are shown in Figure 1.

Among **421 men** with prostate cancer, only **64 (15.2%)** were diagnosed with localized disease, while **110 (25.8%)** had locally advanced disease and the majority, **247 (58.4%)**, presented with metastasis. Most patients were between **55 and 65 years (174, 41.3%)** and **66–75 years (157, 37.3%)**, with just **27 (6.4%)** younger than 55 years. Educational attainment was low, with **269 (64%)** having schooling or below and **173 (41%)** belonging to the low‐income category, particularly among metastatic cases (120, 48.5%). Geographically, the **western (160, 38%)** and **eastern (124, 29.4%)** regions predominated. Clinically, adenocarcinoma accounted for **419 (99.5%)** cases. PSA at presentation was frequently high: **168 (40%)** had levels between 21 and 100 ng/ml, while **133 (31.6%)** had levels >100 ng/ml. Gleason grade group 4 (**126, 29.9%**) and grade group 5 (**182, 43.2%**) were most common, with **142 (57.5%)** of metastatic patients in grade group 5.

Treatment intent was curative in **190 patients (45.1%)**, almost entirely in localized and locally advanced stages, while **231 (54.9%)** were treated with palliative intent, including **224 (90.7%)** of metastatic cases. Radical prostatectomy alone was performed in **16 (3.8%)** patients and radical prostatectomy with adjuvant therapy in **73 (17.3%)**. ADT constituted the mainstay of management, delivered either alone (**103, 24.5%**) or with novel hormonal therapy (**190, 45.1%**), particularly in metastatic disease (**189, 76.5%**). Radiotherapy was rarely used (**10, 2.4%**) (Table 2). Treatment completion was achieved in **356 (84.6%)**, though rates were lower in metastatic disease (197, 79.8%). Among the 65 patients with incomplete treatment, the majority were metastatic (*n* = 50), and the most frequently discontinued modalities were hormone therapy (43%) and chemotherapy (41%) (Table S3).

**TABLE 2:** Distribution of characteristics of study participants (*n* = 421).

Characteristics	Total *n* (%)	Localized *n* (%)	Locally Advanced *n* (%)	Metastasis *n* (%)
	421	64 (15.2)	110 (25.8)	247 (58.4)
Age category (in years)				
<55	27 (6.4)	0	7 (6.4)	20 (8.1)
55–65	174 (41.3)	23 (35.9)	40 (36.3)	111 (44.9)
66–75	157 (37.3)	34 (53.1)	48 (43.6)	75 (30.3)
>75	63 (15)	7 (10.9)	15 (13.6)	41 (16.6)
Education				
Schooling and below	269 (64)	40 (62.5)	54 (49.1)	175 (70.8)
College and above	152 (36)	24 (37.5)	56 (50.9)	72 (29.1)
Occupation				
Employed	216 (51.3)	24 (37.5)	48 (43.6)	144 (58.3)
Unemployed	17 (4)	‐	5 (4.5)	12 (4.9)
Retired	183 (43.4)	39 (60.9)	56 (50.9)	88 (35.6)
Unknown	5 (1.2)	1 (1.6)	1 (0.9)	3 (1.2)
Income				
High (>30 374)	103 (24.4)	21 (32.8)	28 (25.4)	54 (21.8)
Medium (11 362–30 374)	137 (32.5)	22 (34.3)	46 (41.8)	69 (27.9)
Low (<11 362)	173 (41)	19 (29.6)	34 (30.9)	120 (48.5)
Unknown	8 (2)	2 (3.1)	2 (1.8)	4 (1.6)
Region				
West	160 (38)	24 (37.5)	43 (39.1)	93 (37.6)
East	124 (29.4)	19 (29.7)	34 (30.9)	71 (28.7)
North	67 (15.9)	12 (18.7)	16 (14.5)	39 (15.8)
Central	56 (13)	7 (10.9)	14 (12.7)	35 (14.2)
North east	12 (2.9)	2 (3.1)	3 (2.7)	7 (2.8)
Foreign	2 (0.48)	0	0	2 (0.8)
Histology				
Adenocarcinoma	419 (99.5)	63 (98.4)	110 (100)	246 (99.6)
Leiomyosarcoma	1 (0.24)	0	0	1 (0.4)
Clinical	1 (0.24)	1 (1.56)	0	0
PSA at the time of diagnosis				
<10	63 (14.9)	26 (40.6)	18 (16.3)	19 (7.7)
10–20	57 (13.5)	16 (25.0)	24 (21.8)	17 (6.9)
21–100	168 (68.4)	21 (32.8)	55 (50.0)	92 (37.3)
101–500	75 (17.8)	1 (1.6)	13 (11.8)	61 (24.7)
501–1000	23 (5.5)	0	0	23 (9.3)
>1000	35 (8.3)	0	0	35 (14.2)
Gleason score				
Grade group 1 (GS = ≤6)	16 (3.8)	13 (20.3)	2 (1.8)	1 (0.4)
Grade group 2 (GS = 3 + 4)	40 (9.5)	17 (26.6)	16 (14.5)	7 (2.8)
Grade group 3 (GS = 4 + 3)	46 (10.9)	13 (20.3)	21 (19.1)	12 (4.9)
Grade group 4 (GS = 8)	126 (29.9)	14 (21.9)	38 (34.5)	74 (30.0)
Grade group 5 (GS = 9, 10)	182 (43.2)	7 (10.9)	33 (30.0)	142 (57.5)
Unknown	11 (2.6)	0	0	11 (4.4)
Intention of treatment				
Curative	190 (45.1)	64 (100)	103 (93.6)	23 (9.3)
Palliative	231 (54.9)	0	7 (6.4)	224 (90.7)
Treatment completion				
Complete	356 (84.6)	59 (92.2)	100 (90.9)	197 (79.8)
Incomplete	65 (15.4)	5 (7.8)	10 (9.1)	50 (20.2)
Treatment modality				
Radical prostatectomy	16 (3.80)	13 (9.31)	2 (1.82)	1 (0.40)
Radical prostatectomy + adjuvant (chemotherapy and/or radiotherapy)	73 (17.34)	12 (18.75)	30 (27.27)	31 (12.55)
ADT	103 (24.47)	9 (14.06)	18 (16.36)	76 (30.77)
ADT intensification with NHT	190 (45.13)	23 (35.94)	54 (49.09)	113 (45.75)
Radiotherapy	10 (2.38)	1 (1.56)	‐	9 (3.64)
Others	29 (6.89)	6 (9.38)	6 (5.45)	17 (6.88)

**FIGURE 2:**
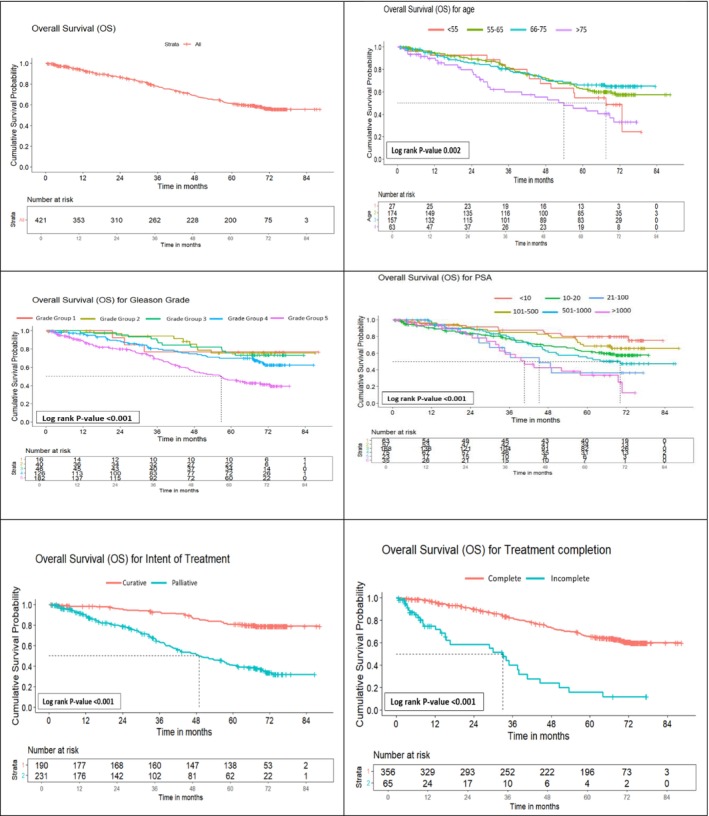
Overall survival by clinico‐epidemiological factors in prostate cancer patients registered at Tata Memorial Hospital, 2017 (*n* = 421).

**FIGURE 3:**
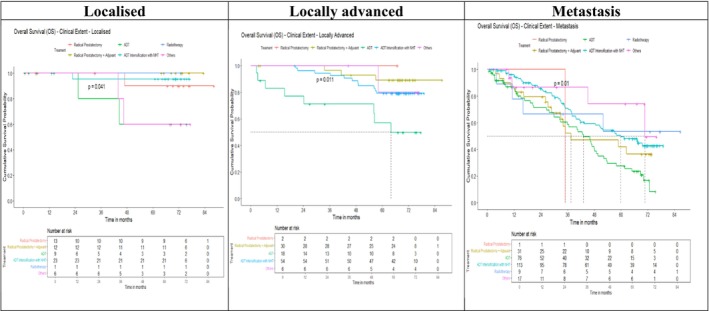
Overall survival by clinical extent and treatment modality (*N* = 421).

The overall 5‐year survival rate in the cohort was 61%, with outcomes strongly influenced by age, PSA, grade and disease extent. Survival decreased with increasing age, from 66% in men aged 66–75 years to 45% in those above 75. Lower Gleason grades and PSA < 10 ng/ml were associated with favourable outcomes (76% and 79%, respectively), whereas grade group 5 and PSA > 1000 ng/ml predicted poor survival (46% and 33%). Clinical extent at diagnosis remained the most important determinant, with 5‐year survival of 89% in localized disease compared to 41% in metastatic cases. Patients completing treatment had markedly superior survival (65% vs. 12%), and curative intent conferred survival benefit over palliative approaches (81% vs. 40%) (Figure 2 and Table S1). Treatment completion varied significantly by age, disease extent, treatment intent and modality, with partial treatment more frequent among older patients, those with metastatic disease, palliative intent and single‐modality therapy, while curative‐intent and combination therapy were associated with higher completion rates (Table S2). Survival patterns by treatment modality further highlighted these differences. In localized disease, radical prostatectomy with or without adjuvant therapy achieved superior outcomes compared to ADT or radiotherapy (*p* = 0.04). In locally advanced disease, multimodal strategies such as radical prostatectomy with adjuvant therapy and ADT intensification significantly outperformed ADT alone (*p* = 0.011). Among metastatic patients, ADT alone yielded the poorest outcomes, whereas intensified or multimodal approaches offered better long‐term survival (*p* = 0.01) (Figure 3 and Table S1).

Multivariate analysis demonstrated that clinical extent, treatment completion and treatment modality were independent predictors of survival. Compared with localized disease, metastatic cases showed a significantly higher risk of death (adjusted HR: 4.59; 95% CI: 1.62–13.02; *p* = 0.004). Incomplete treatment remained a strong adverse factor, with more than a threefold increased risk of mortality (adjusted HR: 3.62; 95% CI: 2.31–5.67; *p* < 0.001). Patients receiving ADT alone had worse survival compared with those on ADT intensification (adjusted HR: 1.65; 95% CI: 1.10–2.46; *p* = 0.01). Radical prostatectomy, with or without adjuvant therapy, and radiotherapy did not show significant survival differences after adjustment. Age and treatment intent were not significant, highlighting the dominant role of disease extent and treatment adherence (Table 3 and Figure S1).

**TABLE 3:** Multivariable Cox regression results showing adjusted hazard ratios for prostate cancer (*N* = 421).

S. no.	Variables	Number	5‐year OS	Unadjusted hazard ratio (95%CI)	*P*‐value	Adjusted hazard ratio (95%CI)	*P*‐value
1	Age category (in years)						
	<55	27 (6.4)	54	0.67 (0.34–1.28)	0.23	0.77 (0.39–1.54)	0.46
	55–65	174 (41.3)	62	0.50 (0.32–0.78)	0.002	0.67 (0.41–1.08)	0.10
	66–75	157 (37.3)	66	0.66 (0.43–1.01)	<0.001	0.74 (0.46–1.97)	0.22
	>75	63 (15)	45	1			
2	Clinical extent						
	Localized	64 (15.2)	89	1			
	Locally advanced	110 (25.8)	79	2.10 (0.85–5.19)	0.1	1.90 (0.74–4.88)	0.18
	Metastasis	247 (58.4)	41	8.89 (3.91–20.2)	<0.001	4.59 (1.62–13.02)	0.004
3	Treatment completion						
	Complete	356 (84.6)	65	1			
	Partial	65 (15.4)	12	3.90 (3.29–7.60)	<0.001	3.62 (2.31–5.67)	<0.001
5	Intent of treatment						
	Curative	190 (45.1)	81	1			
	Palliative	231 (54.9)	40	4.56 (3.12–6.65)	<0.001	1.76 (0.95–3.28)	0.07
6.	Treatment modality						
	Radical prostatectomy	16 (3.80)	84	0.35 (0.85–1.42)	0.14	1.21 (0.27–5.37)	0.80
	Radical prostatectomy + adjuvant	73 (17.34)	71	0.74 (0.44–1.26)	0.27	0.86 (0.51–1.48)	0.59
	ADT	103 (24.47)	35	2.67 (1.86–3.84)	0.00	1.65 (1.10–2.46)	0.01
	ADT intensification with NHT	190 (45.13)	65	Ref			
	Radiotherapy	10 (2.38)	58	1.27 (0.46–1.50)	0.64	0.52 (0.18–1.55)	0.24
	Others	29 (6.89)	71	0.82 (0.38–1.80)	0.62	0.92 (0.42–2.01)	0.83

## 4 DISCUSSION

Prostate cancer remains a growing concern among elderly men, particularly in low‐ and middle‐income countries where delayed diagnosis and disparities in treatment access significantly impact outcomes. In our study of 421 patients at TMH, 34% of patients died, while 20% were lost to follow‐up, yielding an overall follow‐up rate of 80%. The observed overall survival at 1, 3 and 5 years was 93%, 77% and 61%, respectively. These findings are comparable to the Asian average (61.9%) reported in a meta‐analysis by Hassanipour et al. but considerably lower than the 97.1% 5‐year relative survival reported by the US SEER programme.^16^, ^20^ Indian studies similarly show wide variation: a hospital‐based study from Mumbai reported a 64% 5‐year survival, while population‐based data from Punjab revealed only 30.3%^14^, ^15^ (Table S4). Such disparities stem from delayed presentation, incomplete treatment and unequal access to oncology services across regions. The relatively higher survival rates in our cohort likely reflect the availability of advanced therapies, multidisciplinary care and structured follow‐up at TMH.

The mean age of participants was 66.5 ± 8.13 years. Age significantly influenced survival (*p* = 0.002), with the highest 5‐year survival observed in the 66–75 age group (66%), and the lowest in patients aged >75 (45%). Although patients <55 had slightly higher survival than the oldest group (54%), 70% of them were diagnosed with metastatic disease, contributing to worse outcomes. Prior studies, including those by Song,^21^ Kimura,^22^ Merrill^23^ and Humphreys,^24^ have similarly reported poor outcomes in younger patients, often due to advanced‐stage disease and potential BRCA1/BRCA2 mutations in men under 65.^25^ Among older patients (>75), comorbidities in 70% may explain reduced survival, independent of cancer‐specific outcomes. These findings emphasize the role of individualized management that considers both chronological and biological ages.^26^

One of the most striking features of our cohort was the median PSA at diagnosis of 45.94 ng/ml. This is extraordinarily high by international standards. In PSA‐screened populations in the United States and Europe, median PSA at diagnosis is typically in the single digits; screen‐detected series report median PSA values in the range of ≈2.8–5.1 ng/ml.^27^, ^28^, ^29^ Even in East Asian countries with limited screening, median PSA values tend to be lower. A Taiwanese cohort with mainly localized cancers reported a median PSA of 11 ng/ml, whereas a cohort of unscreened Taiwanese patients with advanced disease reported 41 ng/ml.^30^ Our median of ~ 46 ng/ml aligns with these latter unscreened, late‐stage figures, underscoring that Indian men are diagnosed with a much higher tumour burden and often advanced disease.

This is further reflected in stage distribution. In our cohort, only 15.2% had localized disease, 25.8% had locally advanced disease, and 58.4% had metastases. National cancer registry data indicate that only 10%–20% of Indian prostate cancer patients present with organ‐confined disease, compared to 75%–85% of patients in the United States and Europe during the PSA‐screening era.^31^, ^32^ Thus, Indian men even in metropolitan cities such as Mumbai are usually diagnosed beyond the window for curative treatment. This contrast highlights how the absence of organized screening and reliance on symptom‐driven diagnosis contribute to India's poorer outcomes.

High PSA values in our study were associated with advanced pathological features. The majority of patients (84%) fell into high‐grade Gleason categories (grade groups 3–5). While the prognostic value of Gleason score is well established, the more instructive point here is that Indian patients present with such high‐grade disease primarily because diagnosis occurs late. Prior comparative studies have shown that Indian men are more often diagnosed with Gleason ≥7 and metastatic disease compared to Western counterparts,^33^, ^34^ a pattern our cohort also reflects.

Treatment strategies in India remain shaped by these diagnostic realities. Our findings align with other Indian studies showing that combined‐modality therapy improves survival even in advanced disease. Shetty et al. reported superior control with ADT plus radiotherapy compared to ADT alone.^34^ However, the predominance of advanced‐stage presentation is more often managed palliatively, with ADT (surgical or medical). In a north‐east Indian study, 84% of metastatic cases underwent bilateral orchiectomy.^35^ Curative treatments such as radical prostatectomy or definitive radiotherapy are feasible only in a minority of patients, largely because so few present with localized disease at diagnosis. This starkly contrasts with Western centres, where the majority of men are eligible for curative therapy and the focus has shifted towards active surveillance for low‐risk cancers.^36^ Importantly, treatment completion emerged as a strong prognostic factor: 84.6% of patients who completed treatment achieved a 5‐year survival rate of 65%, compared to only 12% among those who did not. These findings echo reports by Budukh et al., who showed a sevenfold survival improvement in patients completing treatment.^14^ Ensuring treatment adherence and access remains a critical public health challenge.

Our 5‐year overall survival of 61% is higher than some Indian population‐based estimates but far below outcomes in Western countries. This disparity is largely explained by stage at presentation. When Indian patients are diagnosed early and receive curative treatment, their outcomes are comparable to international standards. However, the overwhelming majority present with advanced disease, limiting survival. This creates a unique paradox: While high‐income countries debate the harms of PSA ‘overdiagnosis’ and overtreatment, India faces the opposite challenge: underdiagnosis and late detection.

Our findings reinforce the urgent need for improved early detection strategies in India. Routine population‐wide PSA screening is not currently recommended, due to concerns of low incidence, overdiagnosis and limited healthcare resources.^8^, ^37^ However, selective or opportunistic PSA testing—particularly in high‐risk men or as part of structured health check‐ups—could shift diagnosis towards earlier stages. Evidence from urban hospitals and private health networks in India suggests that asymptomatic men detected through PSA testing are more likely to have localized disease and therefore better outcomes.^9^ While resource constraints preclude nationwide screening, targeted approaches could provide a middle ground between over‐ and under‐detection.

However, several limitations warrant mention. Excluding untreated patients may restrict the generalizability of findings, especially to underserved populations. The lack of cause‐specific mortality data prevented cancer‐specific survival analysis. As a hospital‐based registry, the study may over‐represent patients with better access to care, thus inflating survival estimates compared to population‐based studies. Additionally, the absence of data on comorbidities, functional status and patient preferences limits adjustment for important confounders. Despite these limitations, the study offers valuable insights into survival outcomes and treatment patterns for prostate cancer in an Indian tertiary care setting.

## 5 CONCLUSION AND RECOMMENDATIONS

Our study demonstrated that nearly two‐thirds of prostate cancer patients survived 5 years, with survival strongly influenced by age, Gleason grade, PSA level and clinical extent. Patients who received curative‐intent and combination therapy, and who completed treatment, experienced significantly better outcomes. These findings underscore the critical importance of early detection, risk stratification, treatment adherence and comprehensive cancer care. Efforts to improve access, reduce socio‐economic disparities and promote awareness must be prioritized to enhance prostate cancer outcomes in India. Policymakers should recognize prostate cancer as a public health priority, and future research should focus on monitoring survival trends and addressing care inequities through targeted interventions.

## AUTHOR CONTRIBUTIONS

Aswathy P. contributed to methodology, validation, formal analysis, investigation, resources, data curation, visualization and writing of the original draft. Sivaranjini Kannusamy was involved in conceptualization, methodology, validation, formal analysisand writing—review and editing. Sandhya Cheulkar contributed to software development and data validation. Monika Lokhande contributed to formal analysis. Amey Oak, Gagan Prakash, Amit Joshi, Vedang Murthy, Santosh Menon and Rajesh Dikshit were involved in methodology and writing—review and editing. Ganesh Balasubramaniam, Pankaj Chaturvedi and Sudeep Gupta made substantial contributions to project administration and supervision.

## CONFLICT OF INTEREST STATEMENT

No conflicts exist.

## Supporting information


**Table S1:** Overall survival based on sociodemographic and clinical factors (n = 421)
**Table S2:** Distribution of characteristics of study participants based on treatment completion (n = 421)
**Table S3:** Distribution of Treatment modality not Completed among partially treated patients by clinical extent (n = 65)
**Table S4:** Prostate cancer survival rates from published studies
**Figure S1:** Forest Plot Showing Adjusted Hazard Ratios from Multivariate Analysis in Prostate Cancer
