# Supplementary material for: Survival outcomes in prostate cancer patients treated at an Indian tertiary care centre
Source: BJUI Compass. 2026 Feb 5;7(2):e70155. doi: 10.1002/bco2.70155 (PMC12877309; doi:10.1002/bco2.70155)
Supplement: Supplementary file 1 — Table S1: Overall survival based on sociodemographic and clinical factors (n = 421) Table S2: Distribution of characteristics of study participants based on treatment completion (n = 421) Table S3: Distribution of Treatment modality not Completed among partially treated patients by clinical extent (n = 65) Table S4: Prostate cancer survival rates from published studies Figure S1: Forest Plot Showing Adjusted Hazard Ratios from Multivariate Analysis in Prostate Cancer [file BCO2-7-e70155-s001.docx]

**Supplementary Tables and Figures**

**Table S1: Overall survival based on sociodemographic and clinical factors (n=421)**

| **Characteristics** | **Number** | **1 yr (%)** | **3 yr (%)** | **5 yr (%)** | **P value** |
| --- | --- | --- | --- | --- | --- |
| **Overall survival** | **421** | **93** | **77** | **61** |  |
| **Age Category (in years)** | | | | | 0.002 |
| <55 | 27 (6.4) | 92 | 75 | 54 |  |
| 55-65 | 174 (41.3) | 94 | 80 | 62 |  |
| 66-75 | 157 (37.3) | 93 | 78 | 66 |  |
| >75 | 63 (15) | 87 | 57 | 45 |  |
| **Gleason grade** |  |  |  |  | <0.001 |
| Grade group 1(GS=<6) | 16 (3.8) | 100 | 84 | 76 |  |
| Grade group 2(GS=3+4) | 40 (9.5) | 100 | 91 | 75 |  |
| Grade group 3(GS=4+3) | 46 (10.9) | 97 | 86 | 73 |  |
| Grade group 4(GS=8) | 126 (29.9) | 96 | 79 | 69 |  |
| Grade group 5(GS=9,10) | 182 (43.2) | 88 | 68 | 46 |  |
| **PSA at the time of diagnosis** |  |  |  |  | <0.001 |
| <10 | 63 (14.9) | 93 | 85 | 79 |  |
| 10-20 | 57 (13.5) | 96 | 82 | 68 |  |
| 21-100 | 168 (68.4) | 89 | 76 | 61 |  |
| 101-500 | 75 (17.8) | 95 | 76 | 52 |  |
| 501-1000 | 23 (5.5) | 88 | 54 | 36 |  |
| >1000 | 35 (8.3) | 93 | 54 | 33 |  |
| **Clinical extent** |  |  |  |  | <0.001 |
| Localized | 64 (15.2) | 100 | 94 | 89 |  |
| Locally advanced | 110 (25.8) | 97 | 91 | 79 |  |
| Metastasis | 247 (58.4) | 89 | 64 | 41 |  |
| **Intent of treatment** |  |  |  |  | <0.001 |
| Curative | 190 (45.1) | 98 | 92 | 81 |  |
| Palliative | 231 (54.9) | 89 | 62 | 40 |  |
| **Treatment completion** | | | | | <0.001 |
| Complete | 356 (84.6) | 96 | 81 | 65 |  |
| Incomplete | 65 (15.4) | 71 | 36 | 12 |  |
| **Treatment Modality** |  |  |  |  | <0.001 |
| Radical Prostatectomy | 16 (3.80) | 100 | 84 | 84 |  |
| Radical Prostatectomy + Adjuvant | 73 (17.34) | 92 | 78 | 71 |  |
| ADT | 103 (24.47) | 85 | 61 | 35 |  |
| ADT intensification with NHT | 190 (45.13) | 97 | 81 | 65 |  |
| Radiotherapy | 10 (2.38) | 70 | - | 58 |  |
| Others | 29 (6.89) | 92 | 87 | 71 |  |

**Table S2: Distribution of characteristics of study participants based on treatment completion (n=421)**

| **Variables** | **Total n (%)** | **Complete treatment**  **n (%)** | **Partial treatment**  **n (%)** | **P value** |
| --- | --- | --- | --- | --- |
|  | **421** | **356** | **65** |  |
| **Age category (in years)** | | | | |
| < 55 | 27 (6.4) | 26 (7.3) | 1 (1.5) | 0.02 |
| 55-65 | 174 (41.3) | 149 (41.8) | 25 (38.5) |  |
| 66-75 | 157 (37.3) | 135 (37.9) | 22 (33.8) |  |
| >75 | 63 (15) | 46 (12.9) | 17 (26.1) |  |
| **Education** | | | | |
| Schooling and below | 269 (64) | 223 (62.6) | 46 (70.8) | 0.21 |
| College and above | 152 (36) | 133 (37.4) | 19 (29.2) |  |
| **Occupation** | | | | |
| Employed | 216 (51.3) | 184 (51.7) | 32 (49.2) | 0.76 |
| Unemployed | 17 (4) | 14 (3.9) | 3 (4.6) |  |
| Retired | 183 (43.4) | 153 (42.9) | 30 (46.1) |  |
| unknown | 5 (1.2) | 5 (1.4) | 0 |  |
| **Income** | | | | |
| High (>30,374) | 103 (24.4) | 90 (25.3) | 13 (20.0) | 0.66 |
| Medium (11,362–30,374) | 137 (32.5) | 117 (32.9) | 20 (30.8) |  |
| Low (<11362) | 173 (41) | 142 (39.9) | 31 (47.7) |  |
| unknown | 8 (2) | 7 (1.9) | 1 (1.5) |  |
| **Region** | | | | |
| West | 160 (38) | 139 (39.0) | 21 (32.3) | 0.36 |
| East | 124 (29.4) | 103 (28.9) | 21 (32.3) |  |
| North | 67 (15.9) | 55 (15.4) | 12 (18.5) |  |
| Central | 56 (13) | 46 (12.9) | 10 (15.4) |  |
| North East | 12 (2.9) | 12 (3.4) | 0 |  |
| Foreign | 2 (0.48) | 1 (0.28) | 1 (1.54) |  |
| **Clinical extent** | | | | |
| Localised | 64(15.2) | 59 (16.6) | 5 (7.7) | 0.005 |
| Locally advanced | 110(25.8) | 100 (28.1) | 10 (15.4) |  |
| Metastasis | 247(58.4) | 197 (55.3) | 50 (76.9) |  |
| **Intent of Treatment** | | | | |
| Curative | 190 (45.1) | 175 (49.2) | 15 (23.1) | <0.001 |
| Palliative | 231 (54.9) | 181 (50.8) | 50 (76.9) |  |
| **Treatment modality** | | | | |
| Single modality treatment | 149 (35.3) | 103 (28.9) | 46 (70.8) | <0.001 |
| Combination therapy | 272 (64.6) | 253 (71.1) | 19 (29.2) |  |

**Table S3: Distribution of Treatment modality not Completed among partially treated patients by clinical extent (n=65)**

| **Treatment not taken** | **Localised (n=5)** | **Locally Advanced(n=10)** | **Metastatic (n=50)** |
| --- | --- | --- | --- |
| Surgery | - | 1(10) | - |
| Radiotherapy | 2(40) | 2(20) | 5(10) |
| Chemotherapy | - | 2(20) | 23(46) |
| Hormone therapy | 3(60) | 5(50) | 22(44) |

**Table S4: Prostate cancer survival rates from published studies**

| **S.No** | **Author** | **Study Period** | **Study Setting** | **Sample size** | **5-year survival** |
| --- | --- | --- | --- | --- | --- |
| 1 | BC Serda Ferror et al | 1994-2018 | Population, Spain | 9846 | 91.30% |
| 2 | B B Yeole et al | 1987-1991 | Population, India | 740 | 35.10% |
| 3 | Ganesh Balasubramaniam et al | 1999-2002 | Hospital, India (Mumbai) | 371 | 64% |
| 4 | FM Evangelista et al | 2000-2016 | Population, Brazil | - | 79.60% |
| 5 | HAV Ardakani et al | 2001-2012 | Hospital, Central Iran | 100 | 54% |
| 6 | David A. Siegel et al | 2001-2017 | Population, US | 31,04,380 | 97.60% |
| 7 | TP Seraphin et al | 2005-2015 | Population, Africa | 1406 | 39.10% |
| 8 | CJ El Khoury et al | 2007-2011 | Population, US | 2,39,613 | 94% |
| 9 | Fereshte Aliakbari et al | 2010-2015 | Population, Iran | 9772 | 82% |
| 10 | Mohammad Aziz Rasouli et al | 2011-2018 | Population, Iran | 410 | 40.70% |
| 11 | Jemal Beksisa et al | 2012-2016 | Hospital, Ethiopia | 137 | 22% |
| 12 | AM Budukh et al | 2013-2016 | Population, India | 171 | 30.30% |
| 13 | Ramnath Takiar et al | 2014 | Population, India | 1495 | 23.90% |
| 14 | Soheil Hassanipour et al | 2018 | Population, Asia | - | 61.90% |

**Figure S1: Forest Plot Showing Adjusted Hazard Ratios from Multivariate Analysis in Prostate Cancer**

**
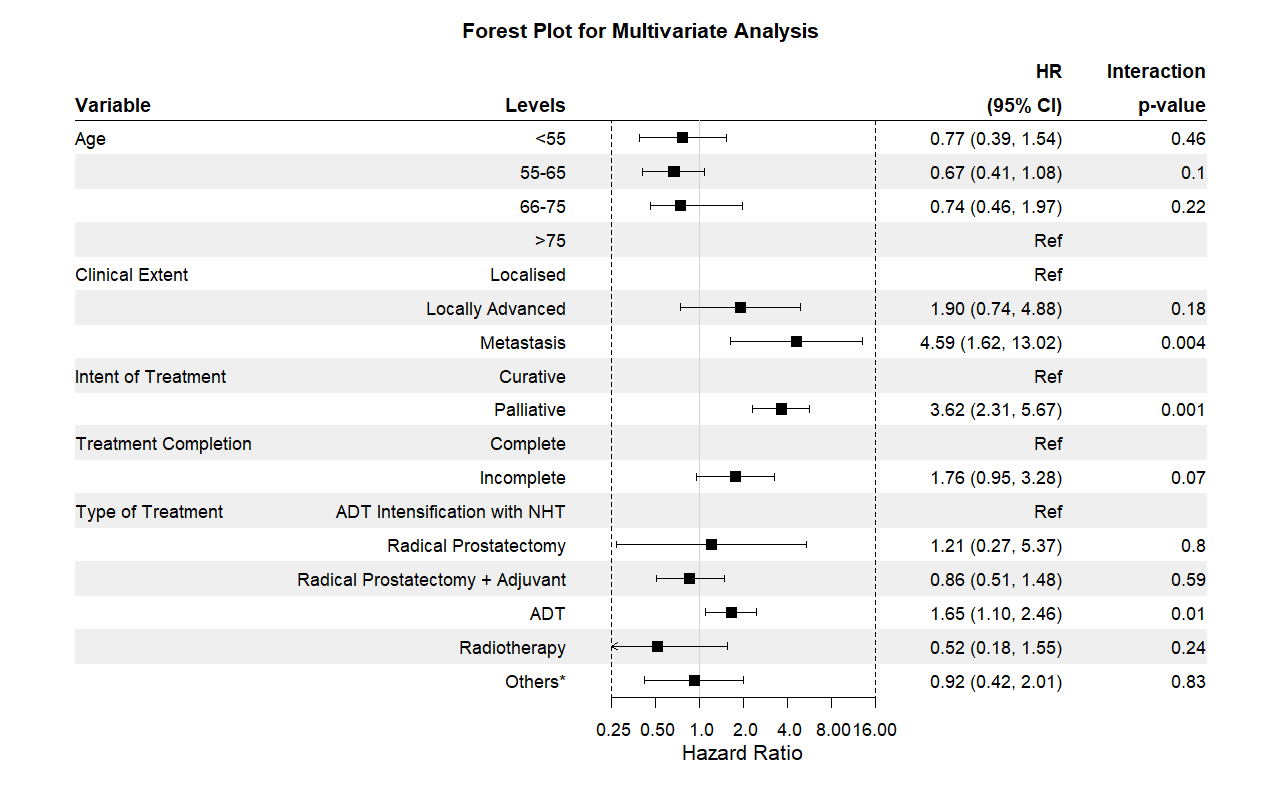
**
